# Chromosome-Level Genome Assembly and Annotation of the Fiber Flax (*Linum usitatissimum*) Genome

**DOI:** 10.3389/fgene.2021.735690

**Published:** 2021-09-13

**Authors:** Rula Sa, Liuxi Yi, Bateer Siqin, Ming An, Haizhu Bao, Xiaoling Song, Shuyan Wang, Zhiwei Li, Zheng Zhang, Hanipa Hazaisi, Jingjing Guo, Shaofeng Su, Jinhuan Li, Xiaoqing Zhao, Zhanyuan Lu

**Affiliations:** ^1^School of Pharmaceutical Sciences, Baotou Medical College, Baotou, China; ^2^Agricultural College, Inner Mongolia Agricultural University, Hohhot, China; ^3^Inner Mongolia Academy of Agricultural and Animal Husbandry Sciences, Inner Mongolia Conservation Tillage Engineering Technology Research Center, Inner Mongolia Key Laboratory of Degradation Farmland Ecological Restoration and Pollution Control, Biotechnology Research Center, Hohhot, China; ^4^Yili Institute of Agricultural Science, Xinjiang, China

**Keywords:** fiber flax, genome assembly, Hi-C, HiFi, genome annotation

## Introduction

Flax (*Linum usitatissimum*), also known as common flax or linseed, is cultivated as an oil and fiber crop in temperate regions and may have been used by humans for as long as 30,000 years (Kvavadze et al., 2009). Fiber flax is one of the primary morphotypes of cultivated flax and the oldest among the domesticated crops and provides a source of fiber for humans (Hickey, 1988). It was reported that disruptive selection for fiber flax (fiber-use) and linseed flax (oil-use) has resulted in plant types that differ considerably in morphology, anatomy, physiology, and agronomic performance (Diederichsen and Ulrich, 2009). Fiber flax is comparatively taller, less branched, and produces fewer seeds than oil-use flax (Zhang et al., 2020). In the last decade, fiber industries developed high-value products for applications in automobile, construction industries, biofuel industries, and pulp (Diederichsen and Ulrich, 2009). Textiles made from flax are known in Western countries as linen and are traditionally used for bed sheets, underclothes, and table linen. Flax remains a minor crop, and the main reason is that its yield has been too low over the last decade (Soto-Cerda et al., 2014).

Accurate reference genomes have become indispensable resources for genetics research, especially for functional gene mapping and marker-assisted selection (MAS). The assembly of the flax genome can significantly accelerate the process of flax breeding. Benefited from the publication of the flax reference genome, quite a few candidate genes related to important agronomic traits were obtained (Soto-Cerda et al., 2018; Xie et al., 2018a,b; You et al., 2018b; Guo et al., 2020). The first flax genome assembly was published in 2012 using Illumina short paired-end and mate-pair reads (CDC Bethune v1) (Wang et al., 2012). Then You and colleagues anchored these fragmented contigs into 15 pseudomolecules using optical, physical, and genetic maps (CDC Bethune v2) (You et al., 2018a). There're also genome assemblies for three different cultivars published recently using short pared-end reads and Hi-C sequencing (Zhang et al., 2020). The first assembly using erroneous long reads for flax was first published a few months ago (Dmitriev et al., 2021). However, the continuity for all these assemblies was very poor, even using the Oxford Nanopore long reads technology. The largest contig N50 for these assemblies was 365 Kb. The flax genome has undergone a very recent whole-genome duplication (WGD) event and is full of repeat elements (You et al., 2018a). It's very prone to collapse between homologous or repeat sequences during the assembly process using short reads or erroneous long reads. It proved this that the assembly sizes varied significantly using different software with Oxford Nanopore long reads (Dmitriev et al., 2021).

The PacBio HiFi reads are produced by calling consensus from subreads generated by multiple passes of the enzyme around a circularized template, resulting in a HiFi read that is both long and accurate. It has been reported that the HiFi technology significantly improved the assembly quality of complex genomes (Chen et al., 2020; Zhao et al., 2021). Many studies have shown that different morphotypes of the same species have large variations in genomes (Song et al., 2020; Guan et al., 2021). In this study, we first combined the HiFi and Hi-C strategies to assemble the fiber flax genome.

## Materials and Methods

### Sample Collection

*Linum usitatissimum* cv. YY5 (Yiya No. 5) is a fiber flax cultivar bred by the Zhang Zheng research team of Xinjiang Yili Institute of Agricultural Sciences. It is a variety bred through an artificial hybridization pedigree selection strategy using Heiya No. 9 as the female parent and 8,738 as the male parent. Its main characteristics are high flax fiber yield (29.20%); mid-late maturity (an average growth period of 89 days); resistance to lodging, Fusarium wilt, and Rhizoctonia solani. The newly sequenced accession used in this study was collected from Inner Mongolia Agricultural University. Seeds were germinated in a light incubator and grew under the circle of 8 h lights at 27°C and 16 h dark at 21°C. Young leaves were sampled for Hi-C and HiFi sequencing after plants grow to 15~20 cm.

### DNA Extraction and Sequencing

High molecular weight genomic DNA was isolated and purified from leaves using Qiagen's MagAttract HMW DNA Kit (QIAGEN, Germantown, MD, USA) following the manufacture's protocol for HiFi sequencing. The resulting HMW gDNA was sheared to a target size of 15~20 kb on the MegaRuptor 3 (Diagenode, Denville, NJ, USA) before library preparation. HiFi sequencing libraries were prepared using SMRTbell Express Template Prep Kit 2.0 (Pacific Biosciences, Menlo Park, CA, USA) and followed by immediate treatment with the Enzyme Clean Up Kit (Pacific Biosciences, Menlo Park, CA, USA). Raw base-called data was processed to generate HiFi reads using the CCS program v4.2.0 (https://ccs.how) with the following settings: minimum pass 3, minimum subread length 50, maximum subread length 50,000, minimum predicted accuracy 0.99.

HiC libraries were created from young leaves, fixed with formaldehyde, and then lysed before the cross-linked DNA was digested overnight with MboI. Sticky ends were biotinylated and proximity-ligated to form chimeric junctions that were enriched for and then physically sheared to a size of 300–500 bp. Chimeric fragments representing the original cross-linked long-distance physical interactions were processed into paired-end sequencing libraries. Paired-end 150 bp reads were generated using the BGI DNBSEQ-T7 platform.

### Genome Assembly

The HiFi long reads were assembled by Hifiasm v0.13-r308 (Cheng et al., 2021) with the default parameters. Then the HiFi reads were mapped back to the assembly to generate a coverage distribution plot using minimap2 2.17-r941 (Li, 2018, p. 2). According to the covering depth, purge_dups v1.2.5 (Guan et al., 2020) was applied to remove redundant haplotigs. The Juicer v1.6 (Durand et al., 2016) and 3D-DNA v180922 (Dudchenko et al., 2017) pipelines were used to process the Hi-C data and scaffold the assembly. The results were polished using the Juicebox Assembly Tools v1.11.08 (Dudchenko et al., 2018). The CDC Bethune v2 assembly has made most use of the long continuity of optical maps. To further improve the accuracy of order and orient in our assembly, we integrated information from the Hi-C scaffolding and the CDC Bethune v2 assembly using the ALLMAPS pipeline (Tang et al., 2015b) implemented in jcvi utility libraries (Tang et al., 2015a).

### Repetitive Element Annotation

We identified repeat sequences of the YY5 v2.0 genome assembly using RepeatMasker v4.1.0. A customer repeat library was constructed using RepeatModeler v2.0.1 (Flynn et al., 2020, p. 2). This pipeline employed RepeatScout v 1.0.6 (Price et al., 2005) and RECON v1.08 (Bao and Eddy, 2002) for *de novo* identification of TEs. Then, high-quality LTR families were discovered using LTRharvest (Ellinghaus et al., 2008) implemented in GenomeTools v1.6.1 (Gremme et al., 2013) and LTR_retriever v2.9.0 (Ou and Jiang, 2018) tools were integrated, following a redundancy removal process. The consensus sequences of TE families were classified using both Dfam v3.1 (Hubley et al., 2016) and Repbase v20181026 (Bao et al., 2015) databases.

### Gene Structure Prediction and Functional Annotation

Gene structure prediction was conducted using an integrative strategy combining protein-based homology searches and transcript data from RNA-Seq of five different tissues, implemented in the Braker2 pipeline v2.1.6 (Hoff et al., 2019; Bruna et al., 2021). First, Viridiplantae proteins from the OrthoDB database v10.1 (Kriventseva et al., 2019) were mapped to the repeat masked genome using ProtHint (Bruna et al., 2020) to generate homologous protein-based hints. RNA-Seq reads were also mapped to the genome using HISAT2 v2.1.0 (Kim et al., 2015). Second, GeneMark-ETP+ collected these two sources of hints for initial unsupervised gene prediction. Then, AUGUSTUS v3.4.0 (Stanke et al., 2006) uses predicted genes for training and then integrates RNA-Seq reads and homologous proteins mapping information into final gene predictions. UTRs were predicted using GUSHR v1.0.0 from RNA-Seq coverage information.

We used six public database resources to conducted gene functional annotation. eggNOG 5.0 (Huerta-Cepas et al., 2019), GO (Gene Ontology Consortium, 2021), and KEGG (Kanehisa, 2002) databases were searched using eggNOG-mapper v2 (Huerta-Cepas et al., 2017) online service. Pfam database v33.1 (Mistry et al., 2021) was searched using the InterProScan v5.50 (Jones et al., 2014) program. Swiss-Prot (Bairoch and Apweiler, 2000) and NR database were searched using DIAMOND v2.0.9.147 (Buchfink et al., 2015) with parameters “–more-sensitive -p 64 -e 1e-6 –max-hsps 1 -k 1 -f 6.”

### Gene Family Analysis

Gene family analysis was performed using OrthoFinder v2.4.0 (Emms and Kelly, 2015). The single-copy gene families were used to construct a species tree. First, each orthogroup was aligned using MUSCLE v3.8.1551 (Edgar, 2004). All alignments were concatenated to build a maximum-likelihood phylogenetic tree using IQ-TREE v2.0.3 (Minh et al., 2020) with default parameters. Then the species tree was calibrated with the obtained branch lengths and calibration points obtained from TimeTree (Kumar et al., 2017) using r8s v1.8.1 (Sanderson, 2003). CAFE v4.2.1 (De Bie et al., 2006) was used to model the expansion and contraction of orthologous gene families.

### Preliminary Data Analysis

**Figure 1:**
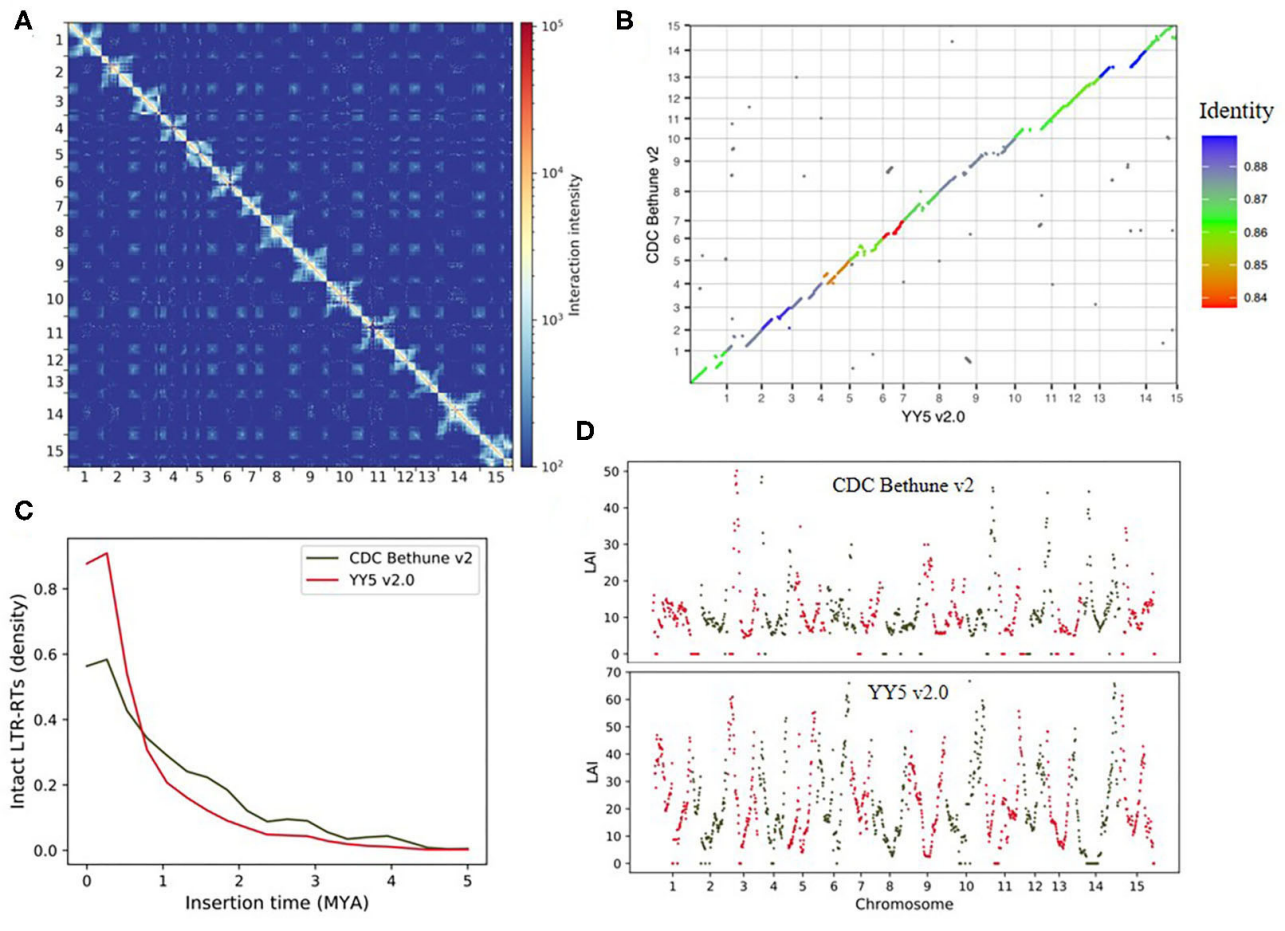
Genome-wide Hi-C interaction heatmaps at 500 Kb windows for the YY5 v2.0 genome assembly **(A)**. Genomic synteny map between the YY5 v2.0 genome assembly and the CDC Bethune v2 genome assembly **(B)**. Estima ted times of insertion for intact LTR-RTs (< = 5 MYA) of the YYS v2.0 assembly and the CDC Bethune v2 genome assembly **(C)**. The YY5 v2.0 and CDC Bethune v2 genome assemblies evaluated by LTR Assembly Index (LAI) **(D)**.

A total of 21.80 Gb HiFi reads were generated with N50 of 12,191 bp and an average pass of 12 (Supplementary Figure 1; Supplementary Table 1). The draft assembly of the HiFi reads has 1,632 contigs covering 537.51 Mb. According to the covering depth, We found a heterozygous peak around 10X depth (Supplementary Figure 2). After removing the redundant haplotigs, an assembly (YY5 v1.0) with N50 of 9.61 Mb and 336 contigs covering 454.95 Mb was obtained. Although the assembly size was decreased by 15.36%, the complete score assessed by BUSCO was improved slightly (2,195 vs. 2,197 for Complete BUSCOs). For Hi-C sequencing, a total of 58.61 Gb high-quality data with a Q20 ratio of 94.6% was obtained (Supplementary Table 2). The Hi-C scaffolding resulted in 15 chromosome-length scaffolds covering 93.0% of the total length (YY5 v2.0, Supplementary Table 3; Figure 1A).

Multiple approaches were used to evaluate the quality of YY5 v2.0 genome assembly (Supplementary Table 3). First, we used BUSCO v4.1.4 (Seppey et al., 2019) to assess the completeness of coding sequences. We identified 94.4% (2197 of 2326) eudicots conserved single copy homologous genes in the genome with the database of eudicots_odb10, which is slightly higher than CDC Bethune v2 assembly (93.5%, 2173 of 2326)(Supplementary Figure 3). Second, The LTR Assembly Index (LAI) (Ou et al., 2018) was calculated to evaluate the assembly continuity of repetitive sequences using the LTR_retriever v2.9.0 (Ou and Jiang, 2018) pipeline. The LAI score of the YY5 v2.0 assembly was much higher than that of CDC Bethune v2 assembly (LAI: 14.29 vs. 9.54, raw LAI: 12.04 vs. 5.47) (Figure 1D), which meets the reference quality, suggested the assembly of repeat sequences of the YY5 v2.0 genome assembly is more complete. From the genome synteny plot (Figure 1B), We can find out that the YY5 v2.0 genome assembly is highly collinear with the CDC Bethune v2 genome assembly except for regions in the central area of chromosomes in which centromere are typically located and full of repeat elements. Sequences are likely missing in the central regions of most CDC Bethune v2 chromosomes compared to the YY5 v2.0 genome assembly. We collected sequences that cannot be aligned with the CDC Bethune v2 genome assembly from the YY5 v2.0 genome assembly. Repetitive elements in unaligned sequences were identified using RepeatMasker v4.1.0 (Smit et al., 2015). We found the unaligned sequences have a higher ratio of repetitive elements than the rest of the YY5 v2.0 genome (86.12% vs. 39.14%), suggesting a more complete assembly of repeat sequences for the YY5 v2.0 genome assembly.

A total of 286,856 EST sequences from *Linum usitatissimum* were downloaded from NCBI and then aligned to the YY5 v2.0 genome assembly using minimap2 with parameters “-t 30 -ax splice -C5 -O6,24 -B4 -uf –secondary=no.” 278,119 (96.51%) EST sequences can be mapped, slightly higher than that of the CDC Bethune v2 genome assembly (96.45%). RNA-Seq reads from five different tissues were also mapped to the YY5 v2.0 genome using HISAT2. The average mapping rate was 94.58% (from 92.62 to 95.44%). Collectively, these pieces of evidence attest to the high quality of our de novo YY5 v2.0 genome assembly, supporting its utility as an excellent reference for genomic variation mining and comparative genome studies in flax.

**Figure 2:**
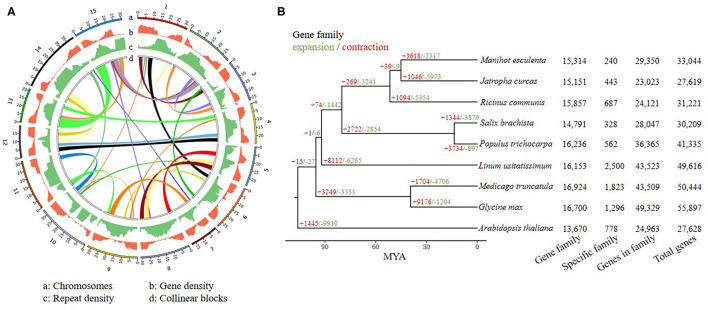
Genomic features and collinear blocks across the YY5 v2.0 genome assembly **(A)**. Gene family characteristics of *Linzan usitatissimum* and eight other dicot plants **(B)**.

A total of 251.86 Mb repetitive elements occupying 55.36% of the YY5 v2.0 genome were annotated (Supplementary Table 4; Figure 2A), including retroelements (26.29%), DNA transposons (9.98%), and other repeats (19.09%). We also re-annotated the repeat sequences of the CDC Bethune v2 genome with the same approach. Only 92.37 Mb repetitive elements occupying 29.21% of the CDC Bethune v2 genome assembly were identified, including retroelements (15.82%), DNA transposons (4.48%), and other repeats (8.91%). The number of intact LTR-RTs identified by the LTR_retriever pipeline in the YY5 v2.0 genome was much higher than that in the CDC Bethune v2 genome (1444 vs. 293). Furthermore, we found that intact LTR-RT insertion events in the YY5 v2.0 genome occurred more recently than that in the CDC Bethune v2 genome (Figure 1C). These clues highlight that our YY5 v2.0 genome assembly provides additional, accurate genome information for chromosomal regions with high repeat sequence content.

A total of 49,616 protein-coding genes and 52,207 transcripts were annotated (Figure 2A). Of the protein-coding genes, 95.3% (2216 of 2326) complete BUSCOs were found slightly higher than that of the CDC Bethune v1 assembly (92.7%, 2156 of 2326, gene annotations for CDC Bethune v2 were unavailable). Among these protein-coding genes, 34,938 (70.42%), 42,697 (86.05%), 22,600 (45.55%), 21,611 (43.56%), 34, 654 (69.84%), and 41,847 (84.34%) genes were annotated with the Pfam, eggNOG, GO, KEGG, SwissProt and NR database separately (Supplementary Table 5). Overall, 43,364 (87.40%) genes were successfully annotated with at least one database.

Gene family analysis was performed for nine dicot plant species, including six Malpighiales (*Linum usitatissimum, Ricinus communis, Jatropha curcas, Salix brachista, Populus trichocarpa*, and *Manihot esculenta*), *Medicago truncatula, Glycine max*, and *Arabidopsis thaliana*. A total of 27,874 orthogroups were identified, including 86 single-copy gene families. 87.7% of flax genes can be assigned to orthogroups. Out of 16,153 orthogroups for flax, 2,500 gene families were specific to flax (Figure 2B). 8,218 genes were contained in these gene families. Then GO and KEGG enrichment analyses were performed (Supplementary Tables 6, 7).

The 86 single-copy gene families were used to construct a species tree. There are 8,112 gene families expanded, and 6,265 gene families contracted in the flax genome compared to the ancestor (Figure 2B). Out of these gene families, 39 significant rapidly evolving gene families involving 592 genes were identified, and GO/KEGG enrichment analyses were also performed (Supplementary Tables 8, 9).

Some of the significantly enriched categories in the flax-specific or rapidly evolving gene families may relate to oil metabolism, fiber biosynthesis, and resistance to biotic stress. The metabolism of pyruvate, aspartic acid, and glutamic acid plays a vital role in the elongation of cotton fiber cells (Ruan et al., 2001). Brassinosteroid biosynthesis promotes the elongation of cotton fiber cells (Ashcraft, 1996). Lipid metabolism plays an important role in the rapid elongation of cotton fiber, and the lipid transport protein gene is preferentially expressed during the rapid elongation period of cotton fiber (Orford and Timmis, 2000). Very-Long-Chain Fatty Acid Synthesis was involved in Arabidopsis cell elongation (Zheng et al., 2005). The metabolism of fructose, starch, and sucrose can promote the formation of cellulose and hemicellulose in the rice stem, thicken the stem wall, enhance its elasticity, and then enhance its lodging resistance (Ishimaru et al., 2008). The biosynthetic pathway of unsaturated fatty acids controls and regulates oleic acid and linoleic acid content in oil crops such as rape, peanut, and soybean (Li et al., 2007). The intermediate products of the phenylpropane metabolic pathway, phenolic substances, and end products of flavonoids, isoflavonoid, lignin, and other substances participate in the process of plant resistance to the invasion of pathogenic bacteria, thereby preventing the infection of pathogenic organisms (Mohib et al., 2018).

## Conclusion

Based on HiFi and Hi-C sequencing data, we assembled a chromosome-scale high-quality genome of the fiber flax YY5. Compared with the previous genome assembly of flax, our assembly quality has dramatically improved, especially improved the assembly of repeating areas. It was proved that HiFi technology is a promising strategy for assembling complex genomes like flax undergone a very recent whole-genome duplication event and is full of repeat elements. We also well-annotated 49,616 protein-coding genes and 52,207 transcripts. Gene family analysis revealed that the specific and rapidly evolving orthogroups in the flax genome might relate to oil metabolism, fiber biosynthesis, and resistance to biotic stress. We believe these new resources will promote genetic research and accelerate the genetic breeding process for flax.

## Data Availability Statement

The datasets presented in this study can be found in online repositories. The names of the repository/repositories can be found below: the HiFi and Hi-C sequencing data have been deposited at the GenBank under the project ID PRJNA725636. The RNA-Seq data can be downloaded from the GenBank under the project ID PRJNA725803. The assembly and annotation files are deposited at the Zenodo (https://doi.org/10.5281/zenodo.4872893).

## Author Contributions

RS performed the experiments and led on manuscript preparation, designed, and interpreted the results. LY, XZ, and ZLu designed the study and analyzed the data. LY and JL managed all samples and interpreted the study, while all other authors revised the manuscript and approved the final version.

## Funding

This study was funded by the Support Plan for Young Scientific and Technological Talents plan B of Inner Mongolia Autonomous Region Colleges and Universities (NJYT-19-B38), Inner Mongolia natural science foundation (2020MS03084), High level talents introduction, and scientific research project of Inner Mongolia Agricultural University (NDYB2019-8), Inner Mongolia natural science foundation (2019ZD04), the Leading Talent Project of Grassland Talents in Inner Mongolia Autonomous Region, Autonomous Region College Students Innovation and Entrepreneurship Training Program (202010130009).

## Conflict of Interest

JL was employed by 8omics Co., Ltd. The remaining authors declare that the research was conducted in the absence of any commercial or financial relationships that could be construed as a potential conflict of interest.

## Publisher's Note

All claims expressed in this article are solely those of the authors and do not necessarily represent those of their affiliated organizations, or those of the publisher, the editors and the reviewers. Any product that may be evaluated in this article, or claim that may be made by its manufacturer, is not guaranteed or endorsed by the publisher.
