# Supplementary material for: Chromosome-Level Genome Assembly and Annotation of the Fiber Flax (Linum usitatissimum) Genome
Source: Front Genet. 2021 Sep 13;12:735690. doi: 10.3389/fgene.2021.735690 (PMC8473814; doi:10.3389/fgene.2021.735690)
Supplement: Supplementary file 1 [file Presentation_1.PPTX]

## Slide 1
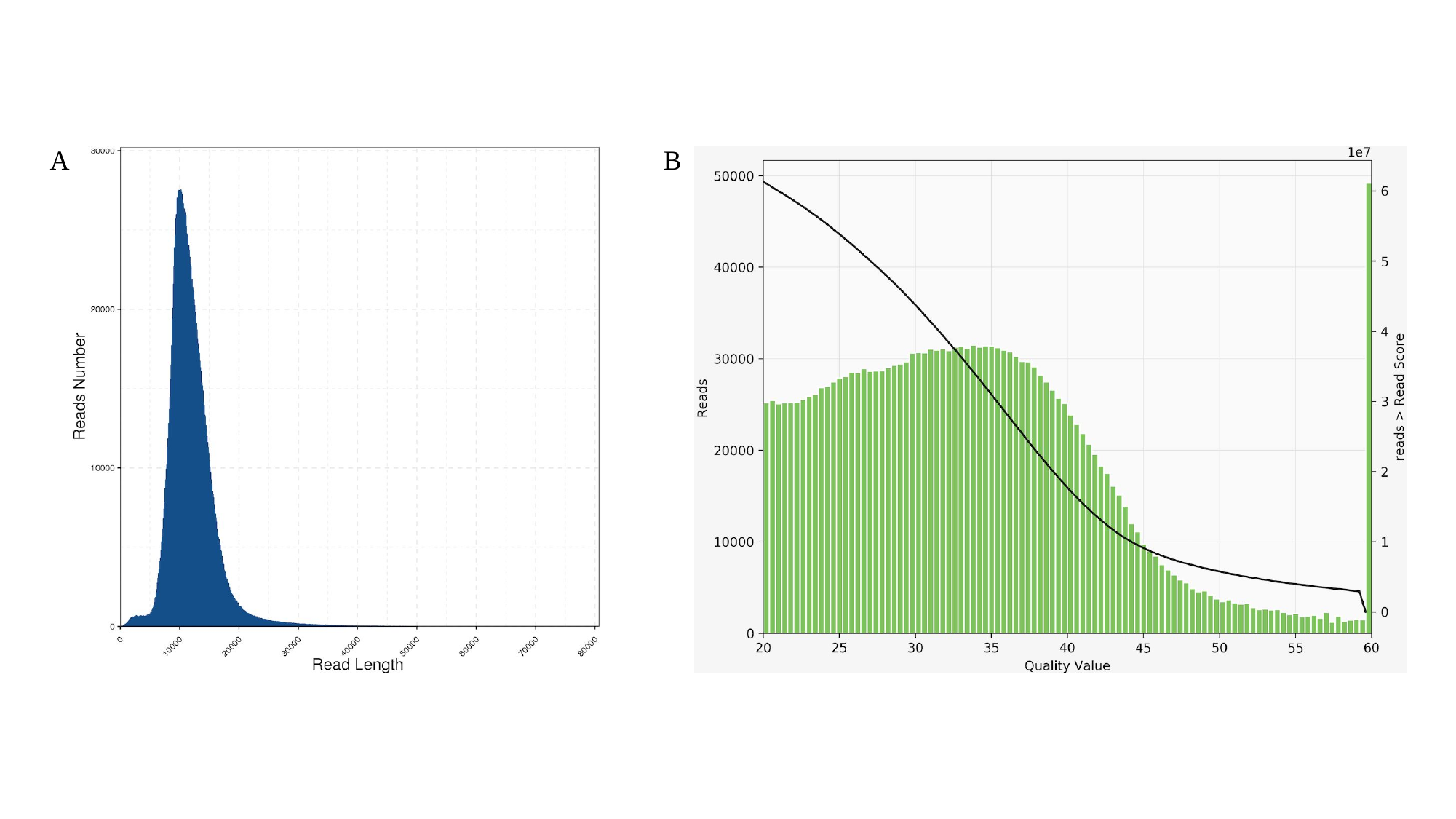

A
B

## Slide 2
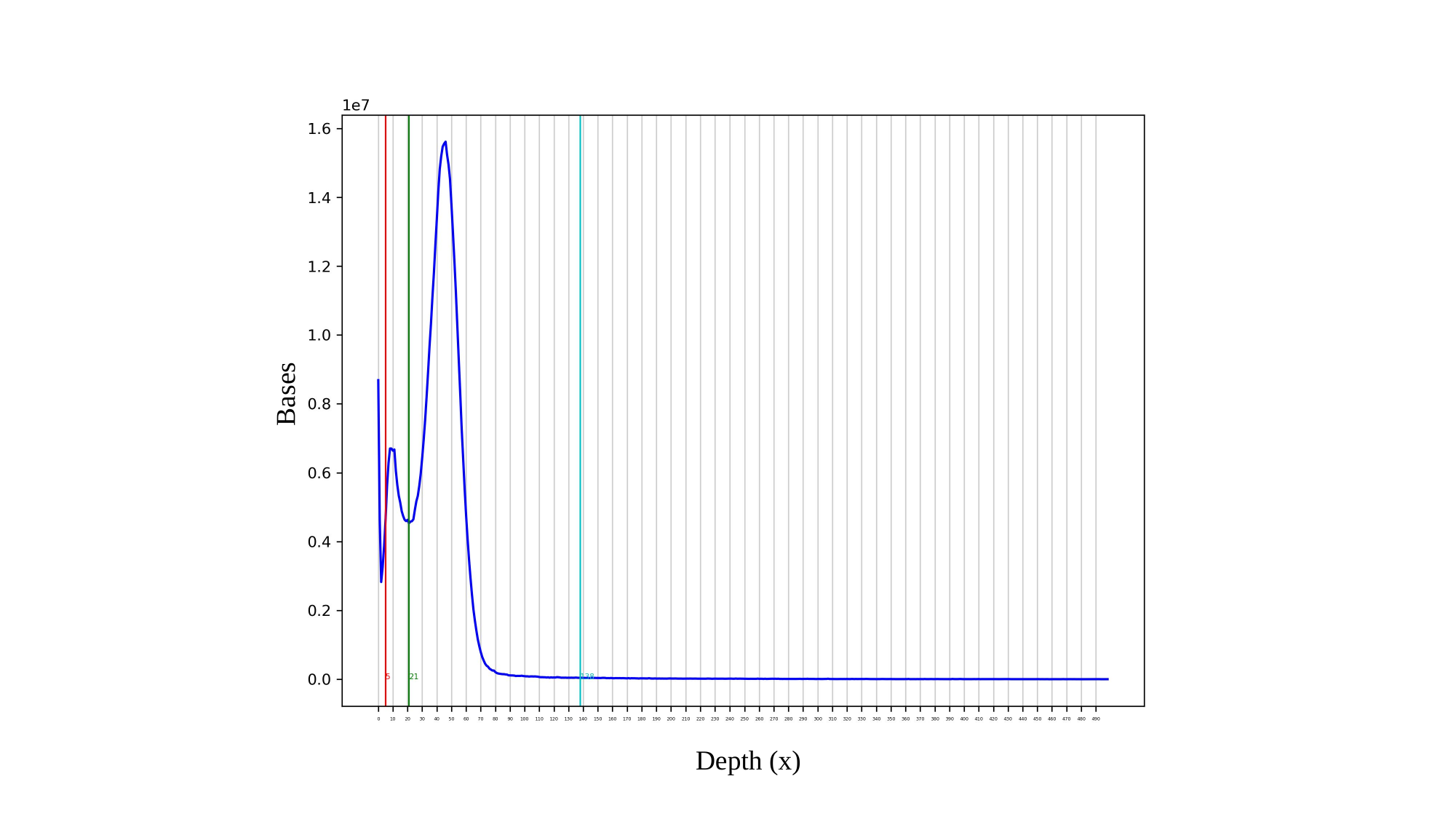

Bases
Depth (x)

## Slide 3
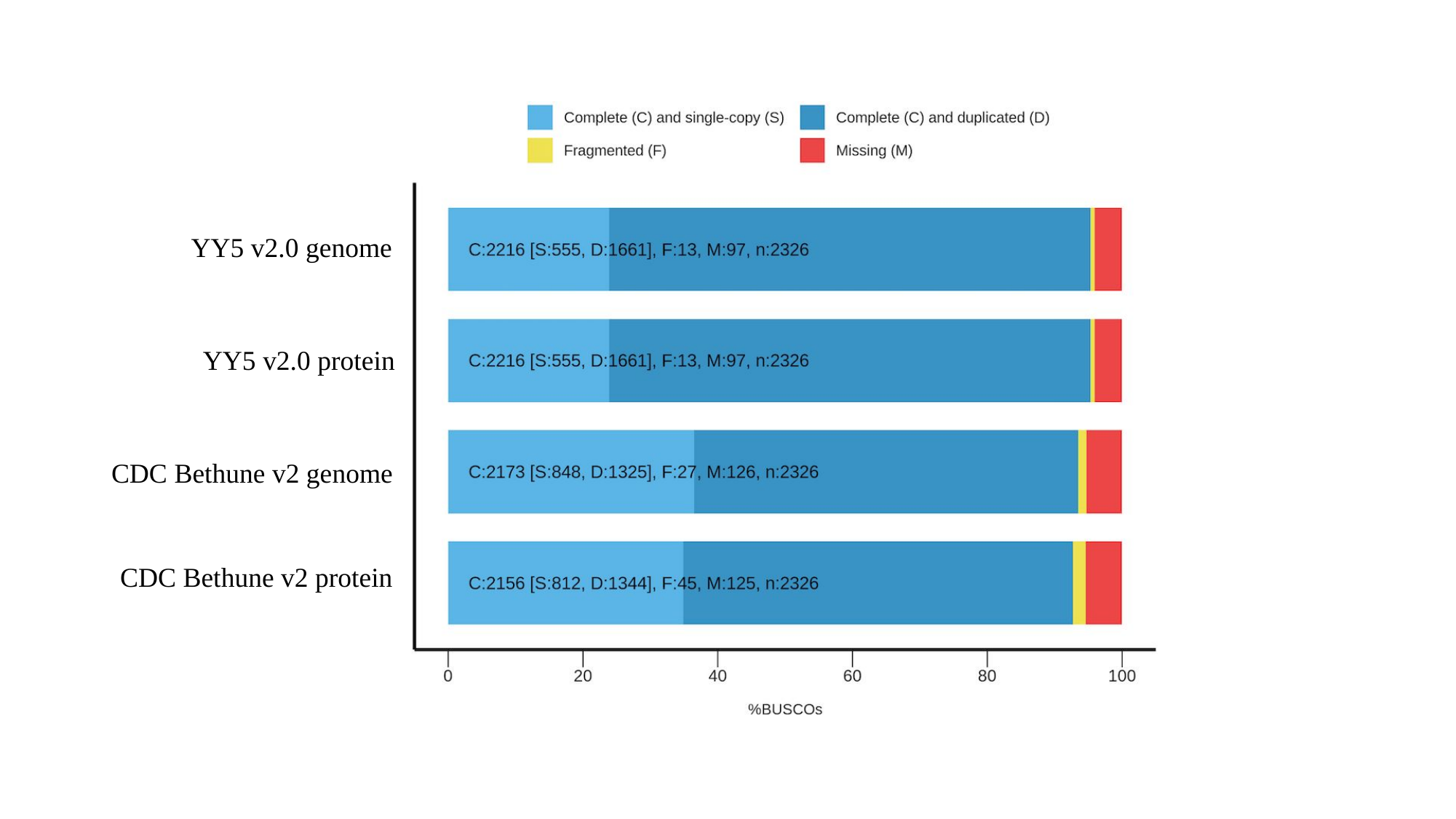

YY5 v2.0 genome
YY5 v2.0 protein
CDC Bethune v2 genome
CDC Bethune v2 protein
